# Synthetic skull bone defects for automatic patient-specific craniofacial implant design

**DOI:** 10.1038/s41597-021-00806-0

**Published:** 2021-01-29

**Authors:** Jianning Li, Christina Gsaxner, Antonio Pepe, Ana Morais, Victor Alves, Gord von Campe, Jürgen Wallner, Jan Egger

**Affiliations:** 1 Institute for Computer Graphics and Vision, Graz University of Technology, Inffeldgasse 16c/II, 8010 Graz, Austria; 2 Computer Algorithms for Medicine Laboratory, Graz, Austria; 3 Department of Oral and Maxillofacial Surgery, Medical University of Graz, Auenbruggerplatz 6/1, 8036 Graz, Austria; 4 Department of Informatics, School of Engineering, University of Minho, Braga, Portugal; 5 Algoritmi Centre, University of Minho, Braga, Portugal; 6 Department of Neurosurgery, Medical University of Graz, Auenbruggerplatz 29, 8036 Graz, Austria

**Keywords:** Translational research, Learning algorithms, Anatomy, Computed tomography

## Abstract

Patient-specific craniofacial implants are used to repair skull bone defects after trauma or surgery. Currently, cranial implants are designed and produced by third-party suppliers, which is usually time-consuming and expensive. Recent advances in additive manufacturing made the in-hospital or in-operation-room fabrication of personalized implants feasible. However, the implants are still manufactured by external companies. To facilitate an optimized workflow, fast and automatic implant manufacturing is highly desirable. Data-driven approaches, such as deep learning, show currently great potential towards automatic implant design. However, a considerable amount of data is needed to train such algorithms, which is, especially in the medical domain, often a bottleneck. Therefore, we present CT-imaging data of the craniofacial complex from 24 patients, in which we injected various artificial cranial defects, resulting in 240 data pairs and 240 corresponding implants. Based on this work, automatic implant design and manufacturing processes can be trained. Additionally, the data of this work build a solid base for researchers to work on automatic cranial implant designs.

## Abstract (summary)

**Table:** 

Measurement(s)	Image Acquisition Matrix Size • Image Slice Thickness • craniofacial region
Technology Type(s)	imaging technique • computed tomography
Sample Characteristic - Organism	Homo sapiens

Machine-accessible metadata file describing the reported data: 10.6084/m9.figshare.13265225

## Background & Summary

Anatomically, the human skull is the skeletal structure of the head and can be divided into two main parts: the neurocranium, which forms the protective cranial cavity that surrounds and houses the brain and brainstem, and the facial skeleton, formed by the bones supporting the face^1,2^. All skull bones are immovably joined together except for the mandible^3^.

Skull bones are made of two types of bone tissue: trabecular bone, which is highly porous, and cortical, which is homogeneous and dense. This porosity within the bone plays an important role in the overall response of the skull to fractures and trauma^4^. The skull is able to protect the brain structures from injury due to its hard unyieldingness, being considered one of the least deformable structures found in nature^5^.

Cranial bone defects can heavily affect a patient’s life quality. Such defects can originate from infections of the calvaria, from heavy craniofacial traumas such as traffic and sport accidents and other physical assaults or occur due to congenital craniofacial deformities or as side effects after neurosurgical or maxillofacial procedures.procedures^6–11^.

According to the Centers for Disease Control and Prevention (2013) from the United States, the majority of craniofacial injuries were because of falls. These are most common among young children (ages 0–4 years), adolescents (15–19 years), and the elderly (over 65 years). Additional causes vary, but prominent among these are automobile and motorcycle accidents^2^. Moreover, subjects with cranial defects might suffer from seizures or visual impairment which makes them even more susceptible to trauma^12^.

Cranioplasty refers to the surgical procedure where a bone defect or deformity in the skull is repaired, which can be achieved through the use of cranial implants^13,14^. The reconstruction of such defects is mostly performed to re-establish the structure and function of the missing cranial bone. A faultless cranial bone is crucial to assure biomechanical protection of the underlying soft tissues of the brain, as well as to normalize intracranial pressure and restore normal cerebrospinal fluid flow dynamics^10,15,16^. In addition, in children patients, an intact cranial vault is crucial for the normal growth and development of the brain^17^.

To ensure the clinical success, cranial implants must fit precisely against all surrounding borders of the bone defect in a way that subsequent movement is avoided, while also successfully restoring the patient’s natural cranial aesthetic shape. In order the cranial implant has to fulfil both functional and aesthetic aspects when used for defect reconstruction^11,12^.

The workflow of the reconstruction of cranial defects with customized patient-specific implants can be divided into four main phases: (1) medical imaging based on radiological data, (2) image processing, (3) computer-aided design and (4) rapid prototyping^18^.

In general, the process starts with acquiring medical 3D imaging data usually from computed tomography (CT) or magnetic resonance imaging (MRI) scans that are converted to Digital Imaging and Communications in Medicine (DICOM) files. Then, the DICOM data is further processed with a medical image processing software to create a 3D model of the scanned defective cranial bone. The 3D model file is then imported into a design software to create the virtual 3D model of the implant, which can, in turn, be manufactured by a Rapid Prototyping (RP) process such as 3D printing^19^. This workflow has shown to be a reliable method for precise craniofacial reconstruction and may even be useful for bone reconstruction at other sites^20,21^.

After obtaining a 3D virtual model of the defective cranial bone, the cranial implant is designed by completely filling the missing part of the skull which corresponds to the defect. However, designing a virtual 3D model of a cranial implant is a challenging task, which often lacks by finding the appropriate software^13^ and needs ample professional experience.

On the one hand, there are commercial software products, such as MIMICS, 3D Doctor and Biobuild. However, these tend to be very license driven and financially expensive and therefore their availability in clinical institutions is usually limited. On the other hand, there are freely available open-source software tools such as Meshmixer and Blender, but since these are not intended for designing medical implants they do not offer convenient tools for this task. Moreover these open-source software tools are not CE-certified for a medical use. Consequently, these tools are not very user-friendly for clinicians to be used during the daily routine and the design process can turn out to be extremely time-consuming^13^. As an alternative to these previously mentioned software products, Egger *et al*.^13^ and Chen *et al*.^6^ proposed CAD software prototypes for the interactive planning and reconstruction of cranial 3D implants. However, these approaches still need some user input and are somewhat time-consuming. Hence, these are also not really an option to be used intra-operatively to prepare a 3D printing process, where a patient would be under anaesthesia until the implant is manufactured. In addition, the effective intra-operative 3D printing time has also to be taken in consideration. Here, a fully automated CAD solution that allows the design process to be less user-dependent and less time consuming, would subsequently be more cost-saving and convenient for both the patient and the clinicians. In that context, a few approaches towards an automatization of computer-aided reconstructions of cranial defects have recently been proposed^22,23^.

Promising approaches consist of creating a reference model and applying a deformation algorithm to approximate it to the desired shape. Fuessinger *et al*.^24^, for example, proposed a data-driven approach for reconstruction of cranial defects based on a SSM (statistical shape model) that captures the shape variability of the cranial vault. Then, the knowledge about this shape variation of healthy patients was used to estimate the missing parts. In this approach, an SSM was generated from 131 craniofacial CT scans of patients with no cranial defects, creating a skull shape template. Then, when performing reconstruction, anatomical landmarks need to be manually selected in the defective skull’s mesh, to establish initial spatial correspondences with the template. Finally, the instance of the SSM that most resembles the anatomical structure of the unaffected parts of the defective skull is retrieved. The resulting shape is an intact cranial vault, very similar to the defective skull’s shape, however without perfectly fitting to the borders of the defect. In order to correct these fitting errors, diffeomorphic deformations are performed to improve the final fit^24^. Unfortunately, to the best of our knowledge, these datasets are not freely accessible. Hence, we provide a CT-based cranial bone image scan series of patients without cranial defects. In these scans, we injected artificial cranial defects into the cranial bones to create training and testing data for algorithms performing automatic cranial defect reconstruction. The image scan series consists of 24 healthy skulls with various artificial cranial defects injected into them, resulting in 240 data pairs, which can be used for training algorithms for automatic cranial implant design. Furthermore the data of this work build a solid base for researchers to work on automatic cranial implant designs.

## Methods

This study was approved by the ethics committee of the Medical University of Graz (MedUni Graz), Austria (EK-30–340 ex 17/18, Medical University of Graz, Austria), trial registration number DRKS00014853. Informed consent have been obtained for this experiment.

**Fig. 1:**
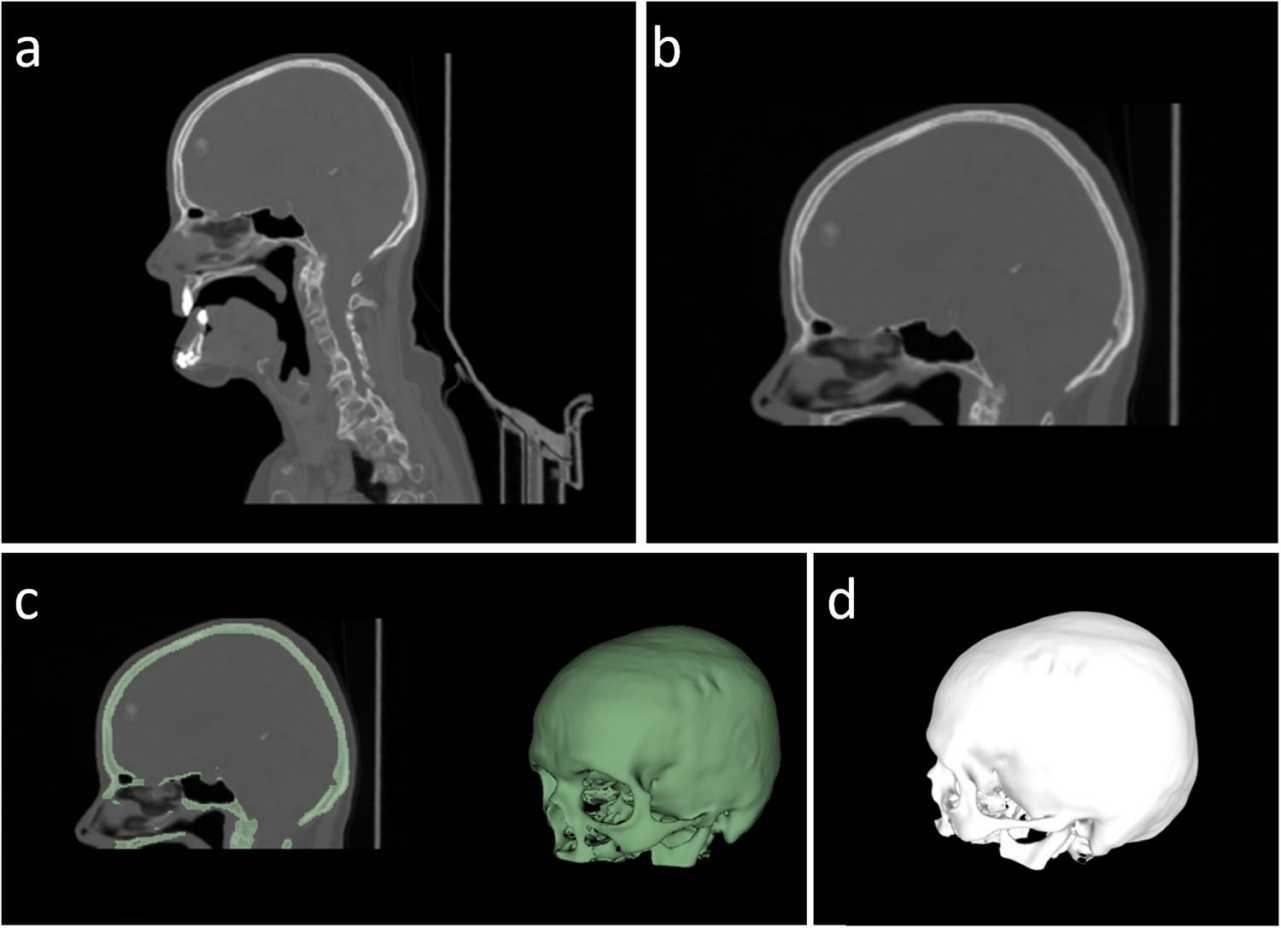
Data processing workflow illustrated using Case02. (**a**) original CT scan; (**b**) CT cropping; (**c**) thresholding (HU value ≥ 150) and CT table removal; (**d**) STL conversion.

Figure 1 shows an overview of the workflow for data collection and preparation. First, clinical CT scans were retrospectively acquired. Second, the head was cropped from the scan. Third, the skull was extracted semi-manually from the CT scans using thresholding and case-specific post-processing strategies. Last, we converted the binary skull map to a 3D model in STL format.

Data processing workflow illustrated using Case02. (a) original CT scan; (b) CT cropping; (c) thresholding (HU value ≥150) and CT table removal; (d) STL conversion.

### Data acquisition and selection

24 skull CT datasets have been collected retrospectively from May 2018 to August 2019 at the Medical University of Graz. CT data were generated according to a standard protocol, using a peak kilovoltage of 120 kVP. Amongst others a Siemens CT scanner (Sensation 64) was used for data generation. The data were originally provided in Digital Imaging and Communications in Medicine (DICOM) format. Only high-resolution CT scans with a voxel resolution of 512 × 512 × Z voxels, where Z ranges from 47 to 490, were selected for our collection. For this work, the original DICOM scans were anonymized by converting them to the Nearly Raw Raster Data (NRRD) format, where all DICOM tags, like patient name, age, sex, etc. are completely removed.

### Skull segmentation

The segmentation of the skulls provided in our dataset were performed semi-manually by experts from the Medical University of Graz using the open-source software 3D Slicer (https://www.slicer.org)^25^.

#### Skull cropping

As a first step, the datasets, which originally covered varying regions of the patient, such as the whole torso or the head and neck area, were cropped to contain the region of the skull only. During cropping, it was made sure to retain all important structures required for cranial implant design, in particular the neurocranium. Most parts of the maxilla and mandible were excluded. An example for original and cropped CT scan is shown in Fig. 1(a,b), respectively.

#### Threshold-based segmentation

Even if the skull can be segmented from CT scans using simple thresholding, it is an experience-dependent task to decide for each CT scan the proper bone threshold, which also requires clinical knowledge in radiologic anatomy. The suitable threshold for each scan was defined in Hounsfield units (HU), which provide the density information of the bone. These can vary greatly with the age of the patient and possible comorbidities (e.g. osteoporosis). The density of calcium deposits must also be taken into account. An overview of the applied values for each case is given in tab:data. The result of this step is a binary map of the patient’s cranium, where a label of one corresponds to structures belonging to the skull, and a label of zero denotes the background.

#### Skull cleaning

Parts of the CT table are sometimes included in the CT scans, which we consider as noise, because it does not belong to the skull bone. Furthermore, in some cases, after thresholding a considerable amount of noise is left inside the skull, which results from the presence of some high density matter in the brain. During thresholding, both the skull and the noise are categorized as foreground, since they have a similar density and, therefore, similar HU values. The high density matter is a calcification within the soft tissue and a reflection of inlays. This is a result of high levels of calcium in the blood (hypercalcaemia), conditioned by bad lifestyle. During an infection or as part of the aging process, calcifications deposit in the soft tissue, including the brain. However, such calcium deposits or calcifications exist in the whole body. The resultant noise can be partly removed by setting the lower bound of the threshold higher, but then also parts of the skull with lower bone density will be removed. Therefore, noise, such as the CT table or speckles from calcifications within the skull were removed by only keeping the largest connected component from the threshold-based segmentation.

Aforementioned calcifications can also lead to uneven surfaces of the segmented skull when they are partly connected to it. Therefore, median smoothing with a radius of 2 mm was applied to those cases. The threshold-based segmentation can also lead to artifacts aside from noise, such as holes in the segmented surface in areas of very delicate, thin bony structures. In case of holes in the threshold-based segmentation, they were filled manually in a slice-by-slice basis. Other artifacts, such as streaking from strongly scattering dental materials, were also manually removed. tab:data shows the applied post-processing strategies for every case. A final, cleaned skull segmentation is visualized in Fig. 1(c).

#### 3D model creation

Triangular meshes and point clouds are the basic data structures in computer graphics. To facilitate the usage of our skull datasets for researchers from the computer graphics field, we also provide 3D triangular meshes of the 24 skulls in the Stereolithography (STL) format with our data collection. The STL mesh is created from the aforementioned binary segmentation map using the open-source software *3D Slicer*. After the binary skull map is generated, we used the’export to model’ functionality under the’Segmentations’ module of *3D Slicer* to create the STL files with default settings. An example of the resultant mesh is shown in Fig. 1(d). Based on the marching cube algorithm, we also provided a python script on Github (https://github.com/Jianningli/SciData) to automatically convert binary skull label maps (in NRRD format) into meshes (in STL format).

### Artificial defect injection

**Fig. 2:**
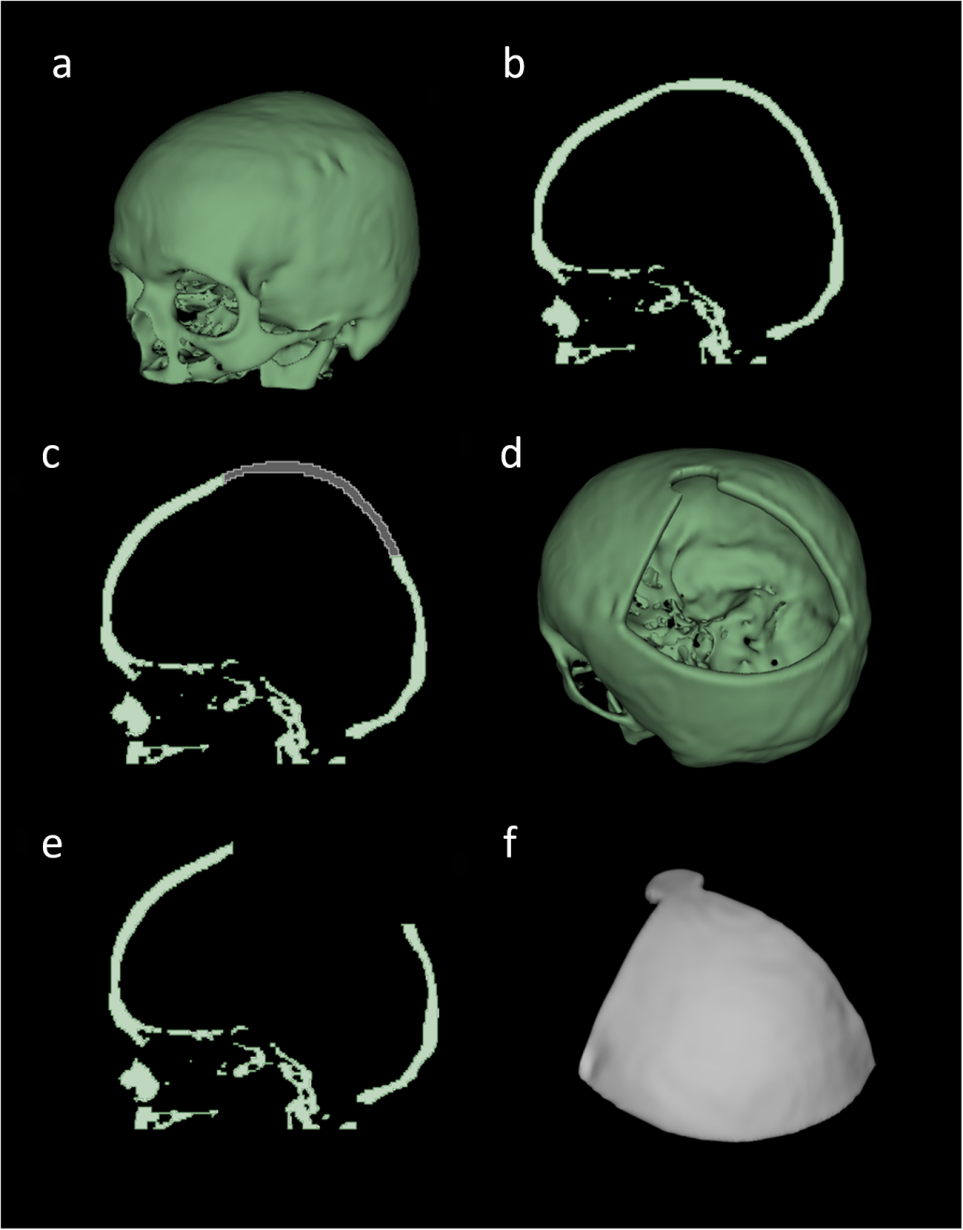
Illustration of defect injection to a healthy skull (Case02). A healthy skull in 3D (**a**) and 2D sagittal view (**b**). The corresponding defective skull in 3D (**d**) and 2D sagittal view (**e**). A portion of the skull (shown in gray) is removed (**c**). The removed portion, i.e., the implant in 3D (**f**).

One of the aims of our data collection is to provide skulls without cranial defects to establish an atlas for cranial defect reconstruction. However, our collection can also be used as training and test set for algorithms reconstructing craniofacial defects. Therefore, we provide software scripts for the injection of artificial cranial defects into the healthy skulls within our data collection. The complete skulls and the skulls with artificially injected defects can be seen as equivalent to skulls obtained from *pre-operative* CT scans and *post-operative* CT scans of patients who had to undergo craniotomy. Figure 2(a,b) shows a healthy skull in 3D (A) and in 2D sagittal view (B). (D-E) show the corresponding defective skull in 3D (D) and 2D sagittal view (E). (F) shows the implant i.e., the portion removed from the healthy skull. (C) shows how the implant should match with the defected region on the defective skull in terms of bone thickness, boundary and shape.

### Artificial defect

The artificial defects in the dataset are realistic but simplified compared to the real surgical defects. The shape, position and size of the real surgical defects do not have a fixed pattern and are determined by the pathological conditions e.g., the size and location of the brain tumor, of each specific patient. However, the real surgical defects do have something in common, i.e., intra-operatively an cranial drill (craniotome) is used in order to open the cranium of the patient and this course of action can leave a small roundish drilling gap on the boarder between the final implant and the defect (clinical implant offset), which does clinically not influence the bone healing process. On the one hand, the virtually created defects are realistic as we mimic the *drilling* process and also include a clinical implant offset, as it can be seen in Fig. 2(d). Additionally, the defect position is randomized and the extent of the defect size is varying. These characteristics of the virtually created artificial defects are also consistent with the real clinical situation and therefore comparably with the clinical practice. On the other hand, the artificial defects are simplified compared to real surgical defects. The border wall of real surgical defects tends to be rough and irregular, due to pathological or surgical reasons. However, the automatically injected defects, as it can be seen in Fig. 2(c), usually have smooth and ‘straight’ border walls.

### Dataset enlargement

**Fig. 3:**
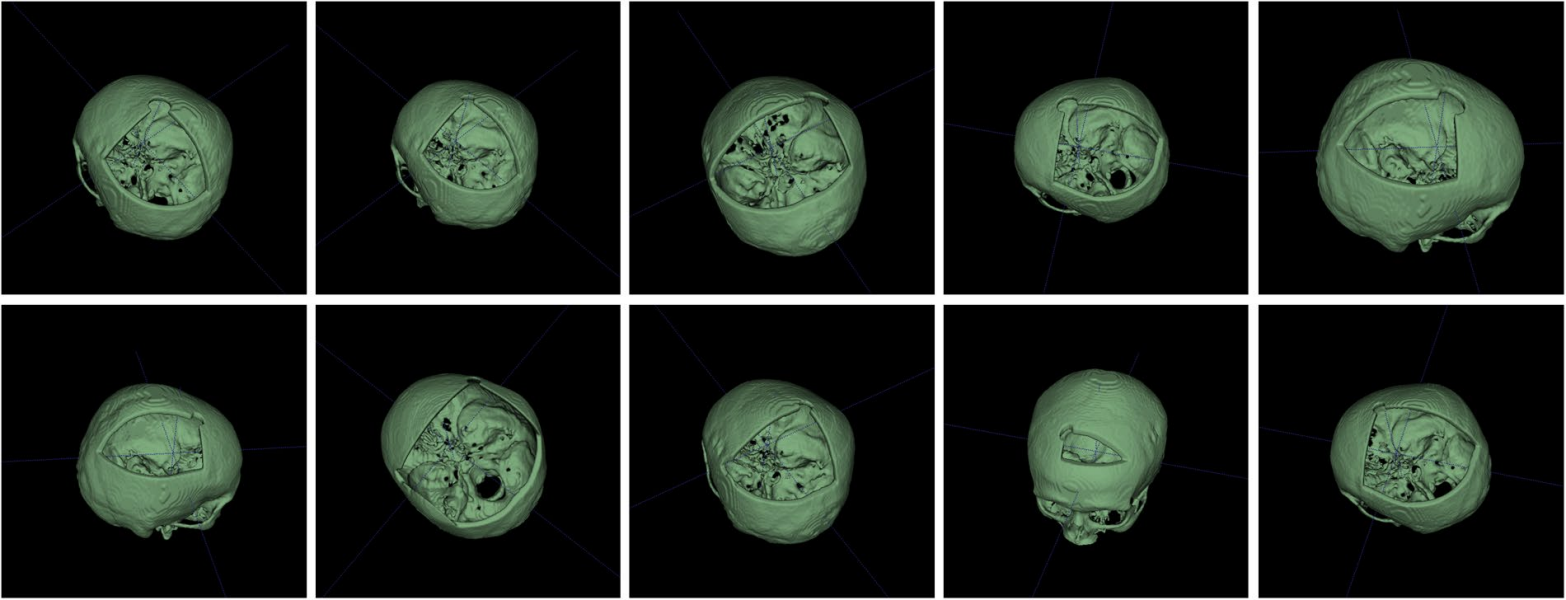
Illustration of ten different defects for Case02.

We create 10 random defects for each skulls. This process also artificially enlarges our dataset, making it applicable to training and evaluation of deep learning algorithms for automatic cranial reconstruction and implant generation. Figure 3 shows the ten defective skulls created out of Case02. It should be noted that even if the artificial defect presented in Fig. 2 does not exactly resemble the defects in a real craniofacial surgery, it demonstrates the feasibility of injecting *realistic* defects for future researchers using these data. Currently, we provide the python scripts for automatic injection of varied defects (e.g., roundish, triangular, rectangular, squarish and etc.) to the complete skull on Github (https://github.com/Jianningli/SciData).

However, there are limitations, when generating a large number of artificial skull defects out of a few without bone defect. This course of action can increase the defect variations but the variations of the skull shape are limited to the original 24 skull bones. If used for training Deep Learning algorithms, the network cannot potentially generalize well to varied skull shapes. Creating multiple defects that are varied in terms of shape, size and position on each skull can increase the defect variations, which therefore can help deep learning algorithms generalize well to varied skull defects. There is currently no strict theoretical nor experimental findings about a quantitative threshold for the enlargement process. However, based on our experience, 10 randomly generated defects tend to be sufficient for deep learning algorithms to learn the defect variations^26^. Generating an excessive high amount of defects, e.g., hundreds or even thousands of defects per skull, is neither efficient nor necessary as excessive defect generation will produce redundant information needed to learn the defect variations. However, as the shape variations of the skull remains unchanged, the deep learning algorithms tend to be overfitting to the 24 skull shapes and can not generalize well on new skull datasets. This can only be overcome by increasing the amount of original cases in a future work. With this work we also hope to inspire other researchers to provide their cases to the research community. In doing so, these additional cases would also cover (different) scanners and scanning protocols from other institutions, thus increasing the variety and making deep learning algorithms more robust.

### Defect injection toolbox

The current toolbox provided on Github (https://github.com/Jianningli/SciData) uses a cubic and spherical mask to ‘erase’ the skull bone that is overlapped with the mask. For each defect generation, the mask moves randomly within the skull to create randomized defects. Depending on where the mask is, the shapes of the resultant defect are not restricted to cubic and spherical, as can be seen from Fig. 3. The real surgical defects tends to be more irregular as it is specific to the pathological condition e.g., the position and size of the brain tumor of the patient. Usually, neurosurgeons use a cranial drill to open the cranium, resulting in a small roundish hole on the corner of the defect. Therefore, the toolbox offers, besides the normal cubic and spherical masks, more realistic defect masks to generate such small roundish holes on the defect corners, as can be seen from Figs. 2 and 3. The current toolbox can be used as a basis and can be extended to generate arbitrarily shaped, positioned and sized skull defects.

## Data Records

The 264 skulls and 240 implants have been uploaded into a figshare repository^27^. Each of the 24 cases is comprised of a NRRD file containing the binary skull segmentation from the corresponding CT scan, a STL mesh file created from the NRRD file as well as a screenshot in Portable Network Graphics (PNG) format of the 3D skull model for a fast preview. Each case is stored in a folder name from Case01 to Case24, and the files associated with the data have the same name within the folder. For convenience, the 240 skulls with artificial defects and the corresponding implants are provided in extra folders. Each case is compressed into a zip file (e.g., *Case01 Defects.zip*, *Case02 Defects.zip* and *Case01 Implants.zip*, *Case02 Implants.zip*). Therefore, for each patient, three zip files are available in the repository, one containing the healthy data, one containing the ten injected defects, and one including the 10 corresponding implants.

### Imaging statistics

Only high quality CT datasets with an in-plane resolution of at least 1.00 × 1.00 mm2 and a slice thickness of maximally 3.27 mm are included within this collection. The dimensions of the 24 original CT scans are 512 × 512 × Z voxels, where Z ranges from 47 to 490. After cropping the scans in axial direction to cover the skull only, the range for Z reduces to 47 to 210. Table 1 summarizes the imaging statistics of the datasets.

**Table 1:** Overview of the imaging statistics of the CT scans within our collection, including parameters and steps used during pre-processing.

Patient	Number of Slices		Inplane Resolution (mm2)	Slice Thickness (mm)	Cropping (yes/no)	Threshold (HU)	Manual post-processing (yes/no)
	whole	cropped					
1	490	87	0.98 × 0.98	2.00	yes	150	yes (artifact removal)
2	244	124	0.98 × 0.98	1.25	yes	150	yes (hole filling)
3	350	148	0.59 × 0.59	1.00	yes	200	yes (smoothing)
4	327	51	0.98 × 0.98	3.27	yes	150	yes (hole filling)
5	286	129	0.98 × 0.98	1.25	yes	150	yes (hole filling)
6	227	123	0.98 × 0.98	1.25	yes	150	no
7	268	144	0.59 × 0.59	1.00	yes	150	yes (hole filling)
8	201	135	0.98 × 0.98	1.25	yes	200	yes (smoothing)
9	175	83	0.98 × 0.98	2.00	yes	150	no
10	327	49	0.98 × 0.98	3.27	yes	150	no
11	47	47	0.98 × 0.98	3.27	no	150	yes (hole filling)
12	242	149	0.41 × 0.41	1.00	yes	150	yes (smoothing)
13	160	124	0.43 × 0.43	1.00	yes	150	yes (hole filling)
14	287	160	0.59 × 0.59	1.00	yes	200	yes (smoothing)
15	231	161	0.46 × 0.46	1.00	yes	200	yes (smoothing)
16	147	101	0.50 × 0.50	1.50	yes	150	yes (hole filling)
17	161	154	0.43 × 0.43	1.00	yes	150	yes (hole filling)
18	318	210	0.49 × 0.49	0.75	yes	150	yes (hole filling/artifact removal)
19	136	78	0.42 × 0.42	2.00	yes	150	yes (smoothing)
20	232	159	0.51 × 0.51	1.00	yes	150	yes (smoothing)
21	168	112	0.46 × 0.46	1.50	yes	200	no
22	200	155	0.46 × 0.46	1.00	yes	150	no
23	110	77	0.45 × 0.45	2.00	yes	150	yes (hole filling)
24	298	195	0.47 × 0.47	0.75	yes	150	yes (smoothing)

## Technical Validation

In^28^, we introduced an automated cranial implant design system in *Studierfenster* (http://studierfenster.icg.tugraz.at/) and elaborated how such a system could potentially be used clinically for cranioplasty. The dataset introduced in this study contains 240 data pairs and can be used for training deep learning models for automatic cranial implant design. In essence, designing a cranial implant is equivalent to generating the complementary piece of a defective skull. Traditionally, the implant is designed semi-automatically with the help of CAD software^6^. The input of such a semi-automatic design workflow is the defective skull and the output is the implant. The deep learning-enabled automated cranial implant design workflow follows similar input/output pattern to the semi-automatic one, i.e., given a defective skull, predict the corresponding complete skull^26,29^. In this sense, the cranial implant can be obtained by taking the difference between the two skulls. Or, given a defective skull, predict the complementary piece directly^29,30^. The deep learning networks used to predict the complete skull or the complementary piece can be trained on the dataset described in this study. The input of the networks is the defective skull and the output is either the complete skull or the implant.

As far as clinical application is concerned, the limitation of the dataset, as mentioned previously, is that the artificial defects are a realistic but simplified version of real craniotomy defect. Deep learning algorithms trained on such dataset may not generalize well to real surgical defects, if not carefully designed. However, the limitation can be overcome algorithmically, i.e., through network design, it is possible to improve the generalization ability of deep learning algorithms. Another straightford approach is to augment the dataset by creating realistic surgical defects on the healthy skulls provided in our dataset. The provided defect injection toolbox can be further extended for realistic defect generation. However, since there is no fixed defect pattern in surgery, it is impractical to consider every possibility during the artificial defect generation process. Improving the generalization ability of the networks through algorithm design is therefore preferred.

## Usage Notes

The data within this work is licensed under the Creative Commons Attribution 4.0 International License (CC BY 4.0). For own research purposes, the data can be freely downloaded and used, but we kindly ask investigators to cite this work in their publications. The data collection provided within this work is free to share. The material can be copied and redistributed in any medium or format. Furthermore, the data can be freely modified - e.g., remixed, transformed, and built upon.

### Data processing

Single skulls in NRRD format from our dataset collection can be visualized and processed using freely available medical imaging software, such as 3D Slicer (https://www.slicer.org), ITK-SNAP (http://www.itksnap.org) or the light-weight web-based medical image visualization and processing platform *Studierfenster* (http://studierfenster.at). 3D meshes in STL file format may be analyzed, manipulated and processed with open source tools such as Blender (https://www.blender.org/), MeshLab (https://www.meshlab.net) or Meshmixer (http://www.meshmixer.com/). Furthermore, the data can also be processed using standard scripting languages such as python (https://www.python.org) and MATLAB (https://www.mathworks.com) with appropriate libraries. Example scrips of processing and visualization of the datasets with python and MATLAB are given in our GitHub repository (https://github.com/Jianningli/SciData). This code can be used as a starting point for researchers wanting to work with our data.

## Data Availability

We provide the python scripts to inject artificial defects to the healthy skulls on GitHub (https://github.com/Jianningli/SciData), which can serve as a starting point for future development based on our skull dataset for other researchers. We also provide additional python scripts for the extraction of point clouds from 3D image volumes and Matlab scripts to convert the triangular, surface meshes of the skulls back to voxel grids (voxelization). The dependencies and usage of the scripts are described in our GitHub repository.
